# Effect of Hot-Rolling Strategy on the Flow Behavior, Productivity, and Mechanical Performance of Ti-6Al-4V Alloy

**DOI:** 10.3390/ma15238344

**Published:** 2022-11-23

**Authors:** Eman El-Shenawy, Hussein Mohamed, Reham Reda

**Affiliations:** 1 Plastic Deformation Department, Metals Technology Institute, Central Metallurgical R & D Institute (CMRDI), Cairo 11421, Egypt; 2 Mechanical Engineering Department, Faculty of Engineering and Technology, Future University in Egypt, New Cairo 11835, Egypt; 3 Mechanical Engineering Department, Faculty of Engineering, Helwan University, Cairo 11732, Egypt; 4 Mechanical Engineering Department, Faculty of Engineering, Suez University, Suez 43512, Egypt

**Keywords:** Ti-6Al-4V alloy, Gleeble simulation, rolling process, flow behavior, productivity, mechanical performance

## Abstract

This work involves studying the effects of applying various designed hot-rolling strategies, using the uniaxial hot compression regimes of the Gleeble 3500 thermo-mechanical simulator on the microstructure, flow behavior, and productivity of Ti-6Al-4V alloy. These strategies were then practically implemented using a rolling mill to produce finished sheets with a thickness of 3 mm. The tensile properties of these finished Ti-6Al-4V sheets were examined, aiming at attaining the optimum rolling strategy conditions that result in upgrading the mechanical performance of the alloy. The undertaken hot-rolling strategies can be divided into two main groups; both comprise applying a total amount of deformation of 75% at a constant strain rate of 0.1 s^−1^. *The first group*, isothermal hot rolling regime (IR), includes three strategies and involves applying the total amount of deformation at constant temperatures, i.e., 900, 800, and 750 °C. *The second group*, non-isothermal hot rolling regime (NIR), includes three strategies and involves partitioning the total amount of deformation into multi-step deformation at variable temperatures in a range of 900–750 °C. The dynamic flow softening is dominant in all IR strategies after the flow stress attains the peak at a low strain value. Then, dynamic flow softening occurs due to the dynamic recrystallization and α phase spheroidization, while a combination of flow softening and hardening takes place on the different passes of the NIR strategies. The designed hot-rolling strategies result in finished sheets with a fine multimodal microstructure that fructifies different mechanical properties that can be employed for different industrial purposes.

## 1. Introduction

Aerospace industries attempt to meet the demanding challenge of reducing production costs, including materials, processing, and labor costs. Moreover, reducing operational consumption, including fuel consumption and maintenance costs, is another challenge. The undertaken research and engineering trend are oriented to satisfy these requirements by manufacturing aircraft parts from materials with a suitable high strength-to-weight ratio [1]. Titanium alloys are extremely suitable and desirable for aerospace applications thanks to their high strength-to-weight ratio, superior mechanical properties, and outstanding corrosion resistance [1,2,3,4,5,6,7,8,9,10,11]. α + β Ti alloys are the most widespread category of Ti alloys [1,5]. The outstanding performance of these alloys deserves to balance the high cost of the material and processing [11]. This category’s mechanical behavior and properties depend highly on its microstructural features, such as size, morphology, and distribution of α and β phases [3,4,5,6,7,8,9,10,12,13]. Therefore, developing and upgrading these alloys mainly depend on the smart design of the processing regime to control the microstructure and properties [11,14]. The most well-known α + β Ti alloy is the Ti-6Al-4V alloy, which is considered an essential material in many industries, such as aircraft, automotive, defense, power plants, and biomedical prosthetics industries [4,5,9,10,11,12,13,14].

In many actual industries, hot working, i.e., hot rolling or forging, is widely utilized to produce semi-finished and finished products [4,11]. To avoid high-cost material loss, metal-cutting operations are typically avoided or minimized for these materials [11]. The α + β Ti alloys are available as semi-finished and final products. The semi-finished products, such as billets, plates, and bars, are obtained from the as-cast α + β Ti ingot, through homogenization and hot deformation with a 50–60% reduction to breakdown the lamellar microstructure to a globular one [3,8,9,14]. These semi-finished products are then subjected to thermomechanical processing to obtain the final products, which may be compressor discs and blades.

Depending on the service conditions and the required properties, these final products may have different microstructural morphologies, i.e., fully equiaxed, fully lamellar, bimodal, and trimodal (mixed) structures [2,5,6,7,8,15]. The presence of a mixture of the different phase morphologies fructifies an outstanding combination of the properties [5]. This admixture microstructure is generated during thermomechanical processing in the α + β range [5]. The formation of an ultrafine structure is a targeted approach, as it promotes mechanical properties and upgrades the service performance [2]. The features of the final microstructure are mainly based on the initial microstructure and the deformation circumstances, i.e., deformation temperature, deformation magnitude, strain rate, cooling rate, etc. [2,3,6,8,15]. These deformation circumstances and flow behavior must be tailored to attain the optimum microstructural features and mechanical properties aimed at improving the performance of the Ti-6Al-4V final products in the different service conditions [6,8,13].

There are many challenges in the processing of α + β Ti alloys, including a limited working parameter range, high working load, vital surface hardening phenomena, and poor microstructure uniformity in the conventional working procedure [6]. To attain the required properties via tailoring the microstructure, these alloys are hot deformed at relatively low temperatures, but the hot workability of these alloys significantly decreases as the temperature decreases [2]. In addition, Ti alloys show scattering deformation behavior of thin sheets, which is related to their anisotropic elastic and plastic properties. Moreover, Ti alloys exhibit a high degree of springback upon forming due to the high yield strength at low elastic modulus [16]. Thus, titanium alloys are considered challenging to process [2,16]. Therefore, due to the complex influence of processing factors, such as rolling temperature, amount of deformation, cooling rate, and initial microstructures, a massive workload is required to reveal the changes in microstructure and the mechanical properties of the finished products of the hot-rolled Ti-6Al-4V alloy [16].

Utilizing a thermomechanical physical simulator, i.e., Gleeble, the hot compression test is a cost-effective, controlled processing tool. Several studies employed this tool to study the influence of the different processing conditions on the microstructure. Most of these investigations were carried out in a single pass deformation at a constant temperature. The Ti-6Al-4V alloy has been widely studied in terms of its many structural morphologies at different processing conditions, but only a few studies have examined the relationship between microstructural characteristics, flow behavior, and final mechanical properties and performance.

The primary deformation mechanism during the hot forming of α + β Ti alloy is the spheroidization of the α phase [2,5,7,12]. This mechanism is considered a dynamic recrystallization process, resulting in flow softening during deformation [2,14]. Upon spheroidization, shearing of α lamellae occurs at a specific strain value. During shearing, dislocations are created, forming interfaces. The migration of these interfaces occurs by diffusion and results in the formation of spheroid grains [2]. Tailoring this mechanism is a significant purpose, as it results in forming a fine equiaxed α + β structure for the mill products [2]. Zhang et al. [2] found that the size and fraction of the spheroid grains increase with the deformation temperature and strain, while they decrease with increasing the strain rate.

Jyoti et al. [8] studied the hot working manner of lamellar and equiaxed initial microstructures of Ti-6Al-4V alloy through a set of hot compression tests, using a Gleeble physical simulator at a constant temperature in a range of 750–950 °C, at a wide range of strain rates and for 60% deformation. They found that a lamellar microstructure exhibits lower activation energy, a higher average flow softening index, and a lower flow stress at later deformation stages than an equiaxed microstructure. They stated that the lamellar morphology is subjected to more softening than the equiaxed one.

Zhang et al. [7] studied the effects of hot compression and post-heat treatment on the microstructure of hot, isostatically pressed Ti-6Al-4V alloy. They reported that the main spheroidization mechanisms in the α + β field at temperatures in a range of 850–920 °C are bending or kinking and fragmentation of the lamellar α phase, whereas, near the β field, at a temperature of about 940 °C, dynamic recrystallization occurs, resulting in the formation of equiaxed α grains along with acicular α precipitates. They found that the fraction and size of the recrystallized grains increase as the strain rate decreases. They also found that the microhardness variation in the specimen deformed in the α + β field is stable, while the specimen deformed in a near-β field shows a considerable variation in microhardness values. This is attributed to the formation of a heterogeneous microstructure after processing and cooling from a near-β field.

Liu et al. [13] studied the effect of multi-pass deformation parameters on the microstructure and flow attitude of hot isostatically pressed Ti-6Al-4V alloy. They performed one, two, and three deformation passes at 950–900–850 °C, at 0.01–1 s^−1^ and different strain values. They found that increasing the deformation temperature and decreasing the strain rate reduces the peak value of the flow stress. Their results evidence that the flow stress decreases after attaining its peak value to different extents. They stated that the basic softening mechanisms are dynamic recrystallization of the β phase, spheroidization of the lamellar α phase, and dynamic recrystallization of the α phase in one-pass deformation with 20% strain, two-pass deformation with 40–10% strain, and three-pass deformation with 10–40% strain, respectively.

In another work, Liu et al. [6] studied the effect of multi-pass deformation on the mi-crohardness of hot isostatically pressed Ti-6Al-4V alloy. They found that the microhard-ness increases with the deformation strain increase and that the lamellar morphology effectively contributes to the hardness value.

The Zener–Hollomon (Z) parameter is an expressive factor of the deformation circumstances. It is helpful to depict the influence of forming temperature and strain rate on the flow characteristics during thermomechanical processing [17]. Low values of this parameter indicate incomplete recrystallization, while their high values exhibit complete recrystallization and the start of grain coarsening [11]. Momeni and Abbasi [4] studied the effects of hot deformation at a constant temperature, in a range of 800–1150 °C, at different strain rates, on the flow characteristics in single- and two-phase fields. They found that the rate of flow softening in the single β range shows a direct relationship to the Z parameter, while, in the α + β range, it is independent of the Z parameter.

Guo et al. [18] studied the influence of interpass time during multipass hot torsion testing of Ti-6Al-4V alloy in an α + β range from 2 s to 32 s under continuous cooling cases. They found that less flow softening takes place at longer interpass times. They stated that dynamic transformation of α phase to β phase occurs during deformation and the total amount of α phase consumed during deformation decreases at longer interpass times as a result of a reverse transformation of the β phase to α phase. They reported that during cooling, flow hardening occurs due to decreasing the temperature and increasing the α phase fraction.

Most papers dealt with studying the effect of working temperature and strain rate, usually using single-pass deformation, on the microstructure, flow behavior, or mechanical properties. Few attempts have considered the correlation between them. Away from simulation, few authors dealt with the actual industrial processes and their influence on the mechanical properties of the finished products.

Yumeng et al. [3] studied the influence of hot rolling temperature at a constant temperature, in a range of 840–930 °C with a 78% reduction on the microstructure and mechanical properties of the Ti-6Al-4V alloy. They declared that α grains are elongated below 900 °C and that dynamic recrystallization does not occur. While the fraction of lamellar structure and recrystallized α grains increases as the temperature increases above 900 °C, the microstructure turns from equiaxed morphology to bimodal as a result of the extreme phase transformation and dynamic recrystallization. They found that the influence of the rolling temperature on the mechanical properties depends on the loading direction. Loading along the rolling direction manifests the lowest strength and the highest ductility, while loading along the normal direction offers the highest strength and the lowest ductility.

Abbasi and Momeni [12] studied the effects of hot compression and hot rolling on the microstructure and tensile properties of Ti-6Al-4V alloy at a constant temperature, in a range of 800–1075 °C, with a total reduction of 75%. Their initial microstructure was a lamellar structure. They stated that hot rolling in the α + β range resulted in α spheroidization while the β range led to the formation of coarse β grains, which transform to martensite by quenching. They found that the tensile properties deteriorate with rising the rolling temperature from the α + β range to the β range.

Hu and Dean [11] examined the influence of hot forging and isothermal hot compression tests on the flow properties of Ti-6Al-4V alloy. They stated that hot forming of this alloy at 950 °C, in the α + β range, involves complex deformation mechanisms, including recovery, recrystallization, and phase redistribution. These mechanisms are susceptible to temperature and strain rate. They reported that flow softening is the dominant mechanism at high temperatures and low strain rates, whereas flow hardening is the dominant mechanism at low temperatures and high strain rates.

Several works are still required to attain the optimum processing procedure that results in the best microstructural characteristics, i.e., grain size, morphology, and homogeneity, and the mechanical performance of the Ti-6Al-4V alloy in order to satisfy the increasing market needs in aerospace and military industries.

The traditional hot-forming conditions involve forming at a high temperature (≥900 °C) and low strain rates (≤0.001 s^−1^) [15]. Recent works aim to reduce the production cost by applying more cost-effective forming conditions, i.e., low temperatures at relatively high strain rates. These working conditions also result in structural refinement and, hence, enhancement in the mechanical properties [15].

In the current work, different hot-rolling strategies were designed based on: (i) tailoring the microstructure characteristics to produce fine constituents and (ii) reducing the total processing time on the production line to reduce the total production cost. The target of the current work is to establish a relationship between the rolling strategy for a semi-finished hot-rolled Ti-6Al-4V alloy plate, its flow behavior, and the mechanical performance of the produced sheets as a final product using uniaxial hot compression tests on a Gleeble 3500 and practical hot rolling regimes on a rolling mill. The information and results of the current work can be used as a guide to the industrial sector of α + β Ti alloys to develop the products of this alloy and improve their service performance.

## 2. Experimental Procedure

**Figure 1:**
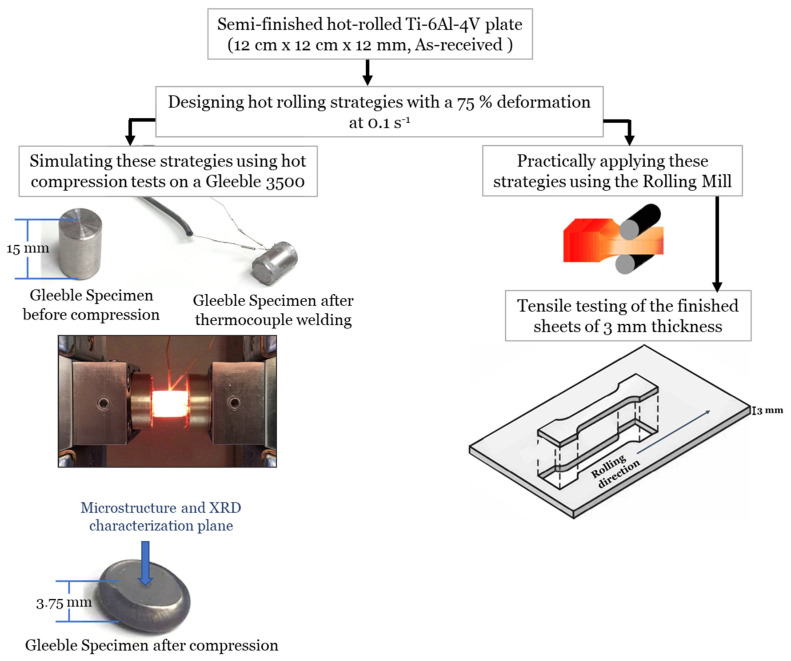
Procedures flowchart.

The current work aims at relating the microstructure characteristics, deformation circumstances, and flow behavior to the final mechanical properties and performance of the Ti-6Al-4V alloy. The undertaken procedures flowchart of the current work is presented in Figure 1.

### 2.1. Materials and Hot Deformation Strategies

As-received material was plates of semi-finished hot-rolled Ti-6Al-4V alloy of 120 mm × 120 mm × 12 mm, supplied by Baoji Intelle Metals Co. Ltd., Baoji, China. The chemical composition of the as-received alloy was assayed using an X-ray fluorescence (XRF) analyzer and presented in Table 1.

**Figure 2:**
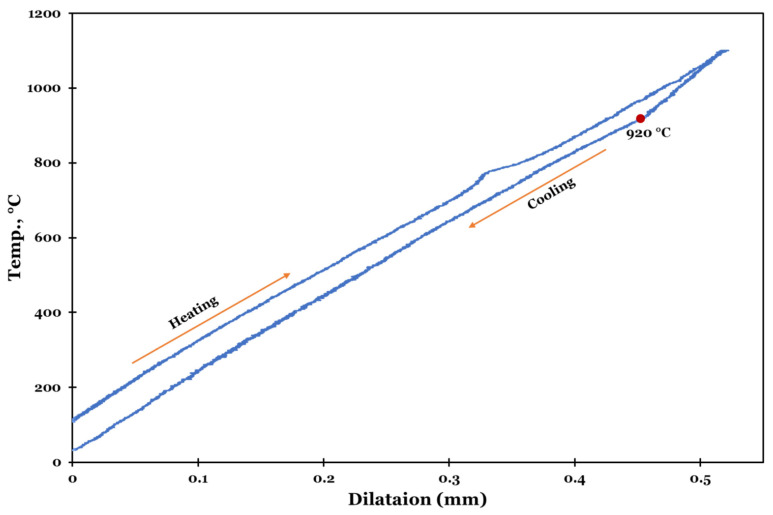
Dilation curve of the as-received Ti-6Al-4V hot-rolled plate.

Initially, the β-transus temperature is detected using a heating-cooling cycle utilizing a thermo-mechanical simulator. The specimen is heated to 1100 °C for 15 min and soaked for 30 s to ensure homogenous temperature distribution, then cooled down to room temperature for 15 min. At the end of this cycle, the change in length of the specimen (dilation) was recorded against the change in temperatures. The dilation test demonstrated that β-transus temperature on cooling is about 920 °C (Figure 2).

The industrial requirements of minimizing the processing time on the production line were considered while designing the hot-rolling strategies. The design idea involves comparing the effects of the same amount of deformation on the microstructure, flow stress, mechanical properties, and processing time if the same amount of reduction is applied in single- or multi-pass deformation.

**Figure 3:**
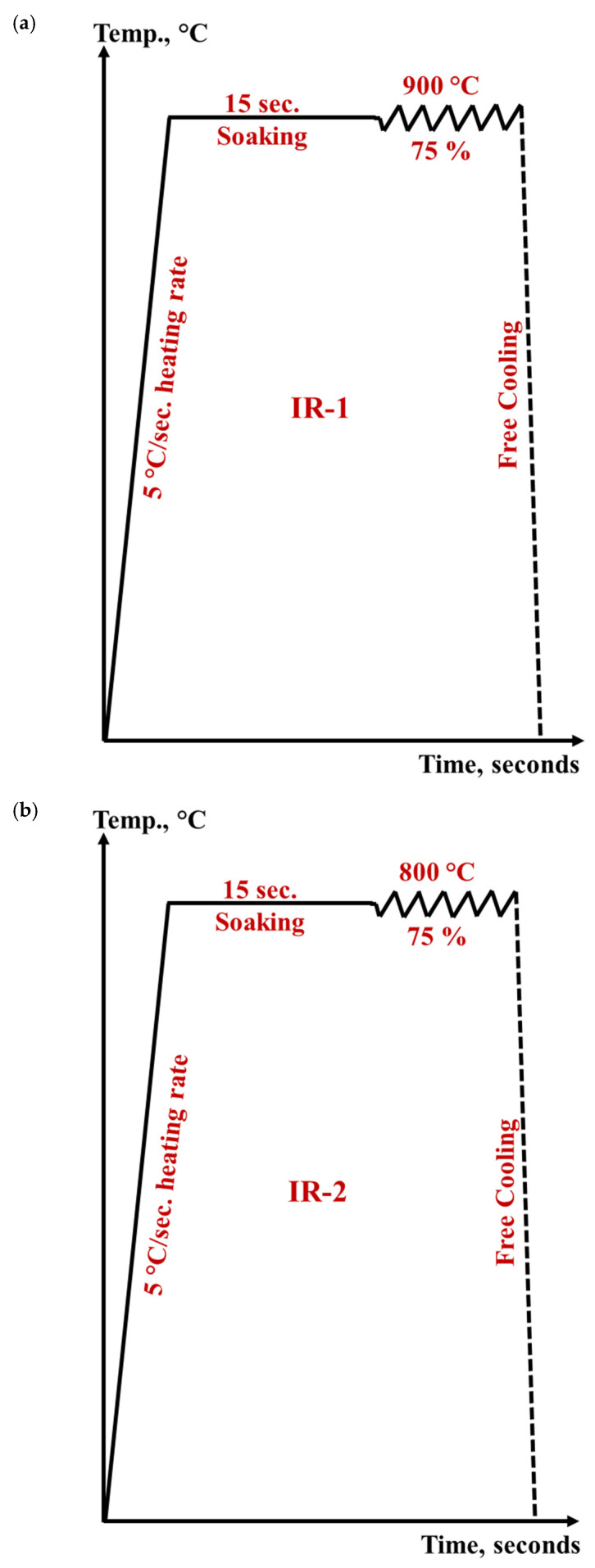

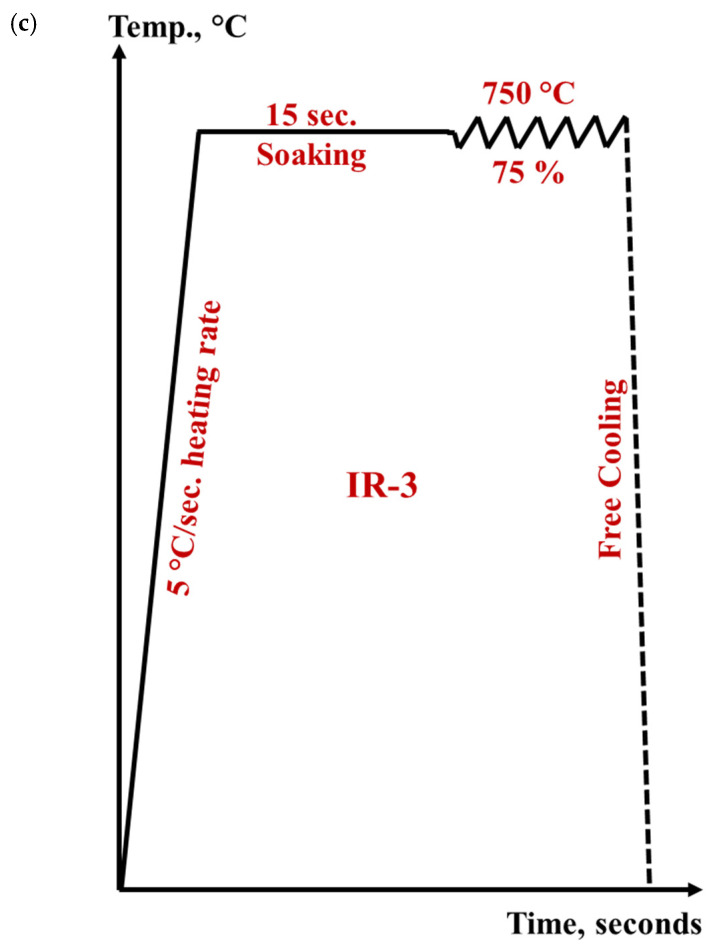
The designed isothermal hot-rolling strategies (IR) at a constant strain rate of 0.1 s^−1^ for 0.75 total strain: (**a**) IR-1, (**b**) IR-2, and (**c**) IR-3.

**Figure 4:**
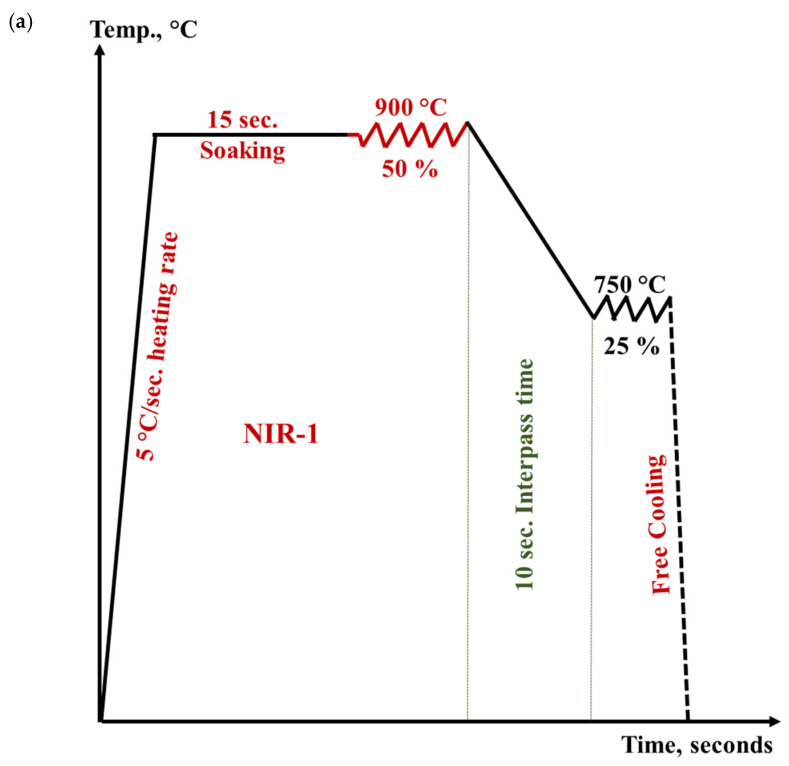

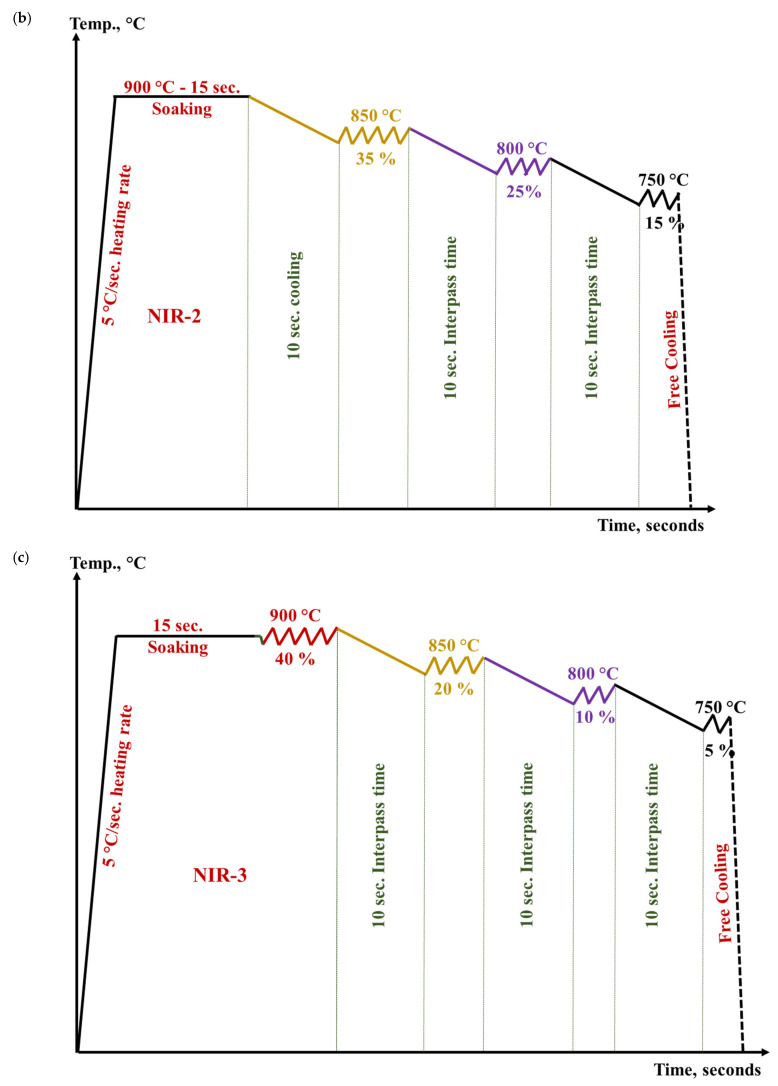
The designed non-isothermal hot-rolling strategies (NIR) at a constant strain rate of 0.1 s^−1^ for 0.75 total strain: (**a**) NIR-1, (**b**) NIR-2, and (**c**) NIR-3.

A series of hot-rolling strategies was performed on semi-finished hot-rolled Ti-6Al-4V alloy with an overall amount of deformation of about 75% at a constant strain rate of 0.1 s^−1^. These strategies can be divided into two groups. The first group is called the **isothermal hot rolling regime (IR)** and comprises three rolling strategies. This group involves applying the total amount of deformation of 75% at constant temperatures, i.e., 900, 800, and 750 °C, which are designated by IR-1, IR-2, and IR-3, respectively. Figure 3 illustrates the schedule of isothermal rolling regimes (IR). The second group is called the **non-isothermal hot rolling regime (NIR)** and includes *three* strategies, designated as NIR-1, NIR-2, and NIR-3. This group involves partitioning the total amount of deformation of 75% into multi-step deformation at variable temperatures (Figure 4). In the NIR-1 strategy, the total amount of deformation is accomplished at two-step deformation temperatures, while in the NIR-2 and NIR-3 strategies, the total amount of deformation is divided into three- and four-step deformation at different temperatures, respectively.

All the designed strategies involve heating with a rate of 5 °C/s up to 850–900 °C and soaking for 15 s to ensure a homogeneous temperature distribution before deformation. All rolling strategies were started at a high temperature in α + β field in order to avoid the grain coarsening and structural heterogeneity that occurs when heating to β field, and reduce the required flow stress and the soaking time. This in turn minimizes the processing cost when these hot-rolling strategies are applied on an industrial scale.

From a cost-side view, a high strain rate is preferable to reduce the processing time. Unfortunately, high strain rates negatively affect microstructural characteristics as they result in lamellae kinking and flow localization, which causes cracking. Therefore, in designing the current rolling strategy, a moderate strain rate of 0.1 s^−1^ was selected to avoid flow localization and reduce the total time on the production line.

Each deformation pass was implemented at a constant temperature in a temperature range of 900–750 °C, followed by cooling. For NIR strategies, an interpass time (delay time) of 10 s was involved in compensating for the required period for transferring the plates from the rough deformation stands to the finish deformation stands. This moderate value of the interpass time results in less softening [18] and reduces the total production time. The post-heat treatment is excluded to reduce the time and cost of production.

### 2.2. Uniaxial Compression Studies—Gleeble Tests

The designed hot deformation strategies (Figure 3 and Figure 4) were executed using uniaxial compression studies of the Gleeble 3500 thermo-mechanical simulator. Specimens for the thermomechanical simulator were machined from the as-received plates, in the rolling direction, into cylinders of 10 mm in diameter and 15 mm in length. Each specimen was then welded to an S-type (platinum-rhodium) thermocouple at the specimen surface using a thermocouple spot welder device and then fixed between two jaws of stainless steel in the thermo-mechanical simulator. After deformation, all the designed strategies have undergone free cooling with a calculated rate of 2 °C/s to 70 °C temperature.

### 2.3. Practical Hot-Rolling Strategies

All the designed hot deformation strategies (Figure 3 and Figure 4) were experimentally implemented on a rolling mill. Air cooling to room temperature was undertaken in the final stage to simulate the actual production practice. The initial thickness of the as-received plate is 12 mm and the final sheet thickness is 3 mm.

### 2.4. Characterization

The as-received and Gleeble hot-deformed specimens were prepared using the standard metallographic procedure that consists of grinding, polishing, and etching in a solution of 10% HNO_3_, 5% HF, and 85% distilled water. The specimens were investigated using optical and scanning electron microscopes (SEM). X-ray diffraction (XRD) was performed to identify the different phases. The average grain size was estimated using image J software.

In addition to the flow behavior, the mechanical properties of the final sheets are also studied. According to ASTM E8, tensile specimens were machined from the as-received plates and practically hot-rolled sheets. Uniaxial tensile tests were performed in the rolling direction at room temperature and with a strain rate of 0.5 mm/s.

## 3. Results and Discussion

### 3.1. Effect of Hot-Rolling Strategies on Microstructure

The response of the microstructure during the different hot-rolling strategies may be of particular interest in designing industrial hot-working schedules during the manufacture of titanium alloys.

**Figure 5:**
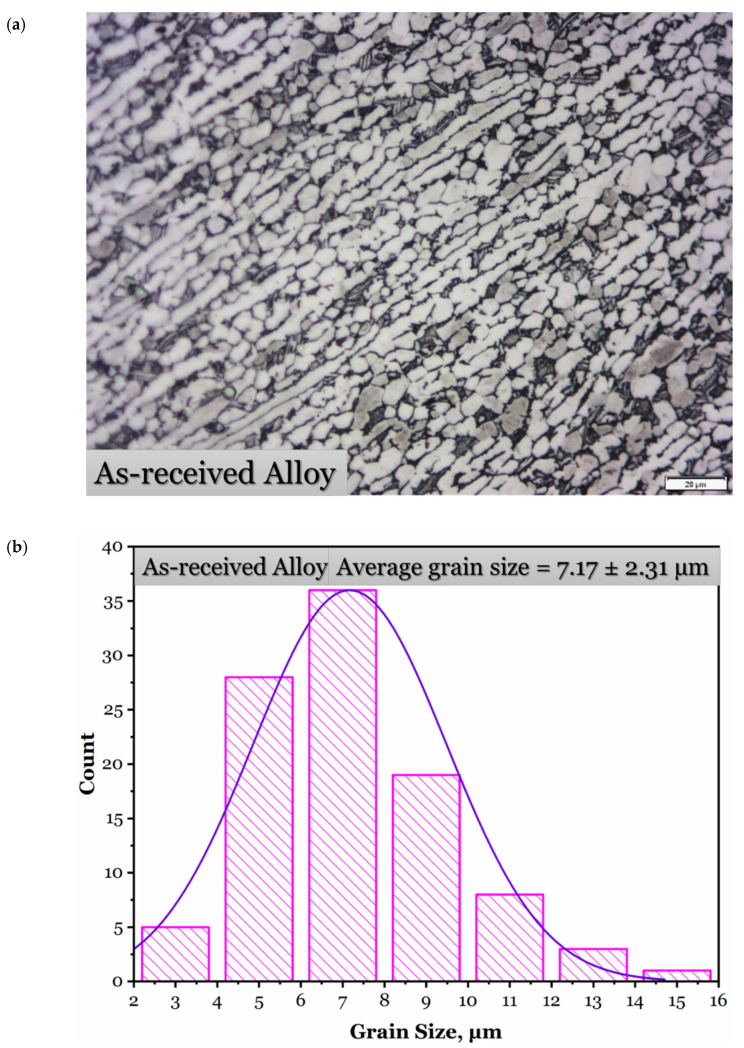
Microstructure of the as-received hot-rolled Ti-6Al-4V alloy (**a**) and a histogram of the grain size distribution (**b**).

The as-received alloy microstructure is composed of a mixture of elongated α phase, equiaxed α + β grains, and tiny islands of acicular α + β structure (Figure 5a), with a relatively heterogeneous distribution of grain size, as demonstrated in the histogram in Figure 5b. The average grain size is estimated to be 7.17 ± 2.31 µm with a β fraction of 9.8%.

**Figure 6:**
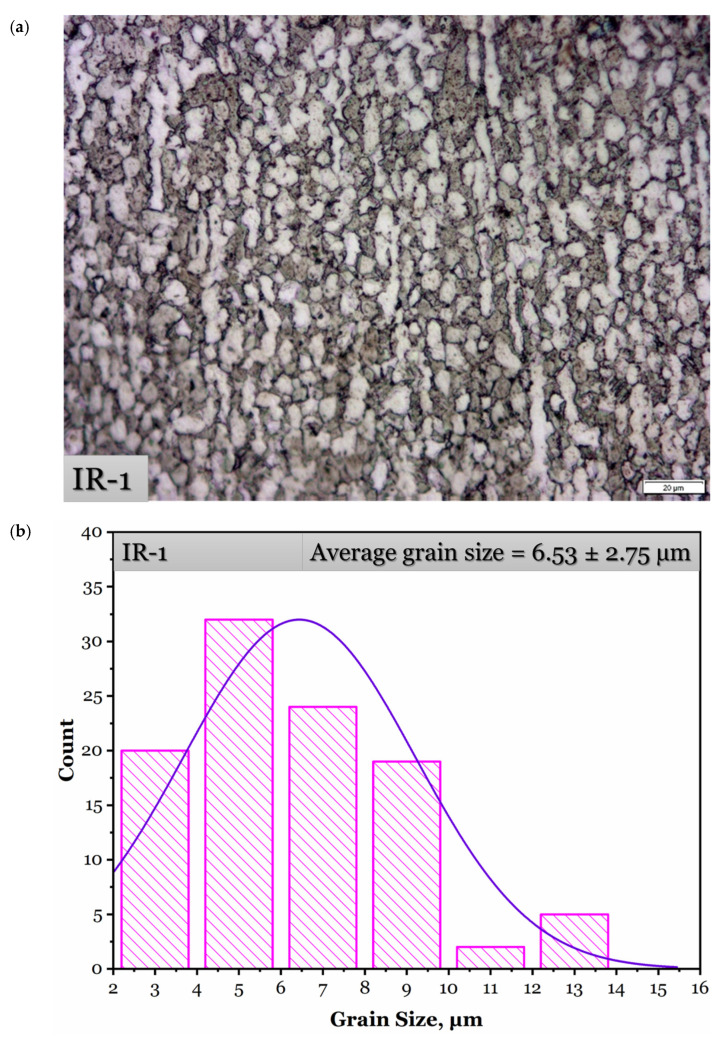

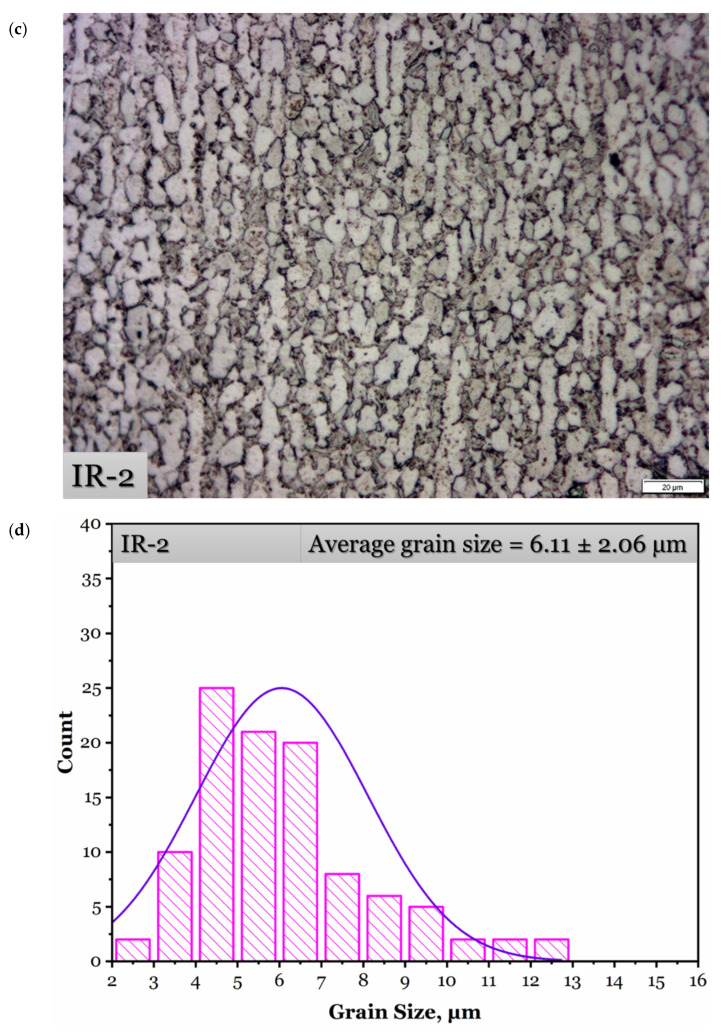

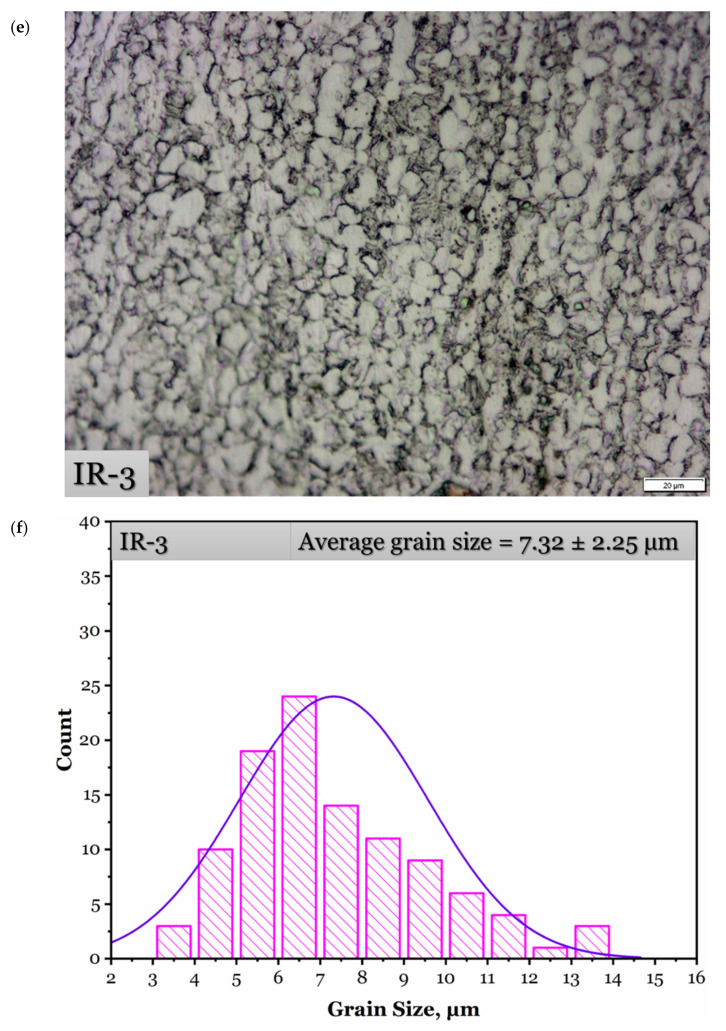
Optical micrographs after isothermal hot-rolling strategies and their histograms of the grain size distribution: (**a**,**b**) IR-1, (**c**,**d**) IR-2, and (**e**,**f**) IR-3.

**Figure 7:**
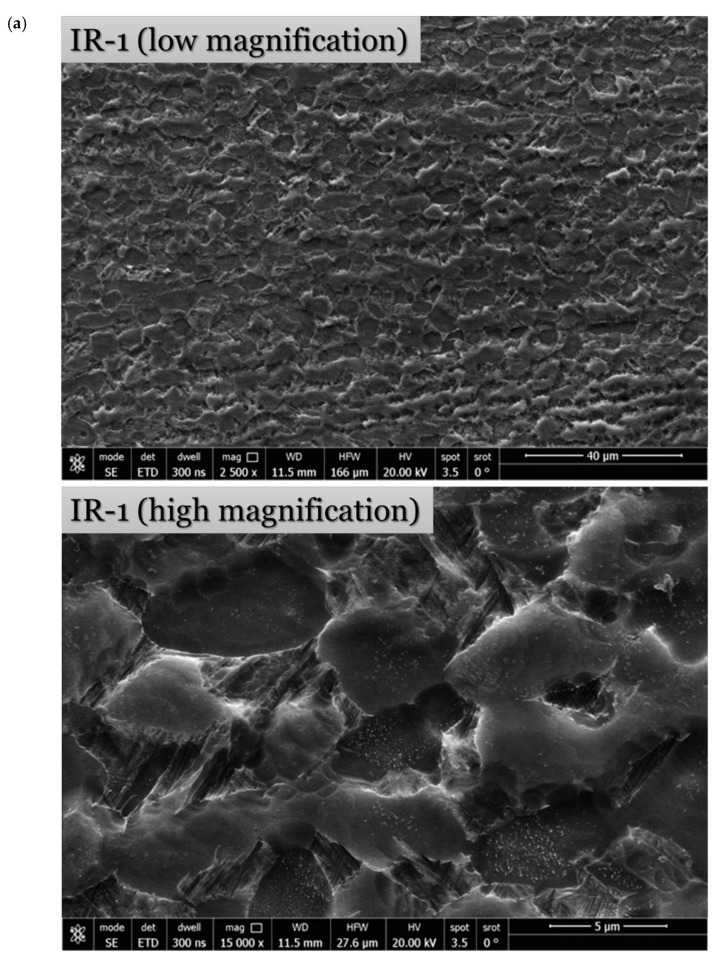

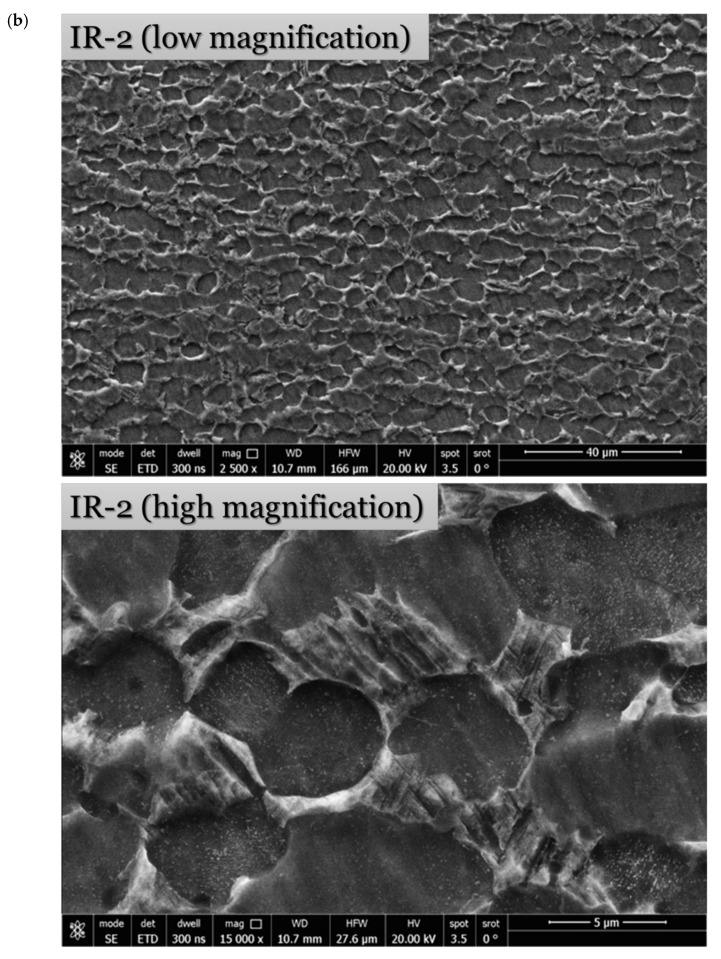

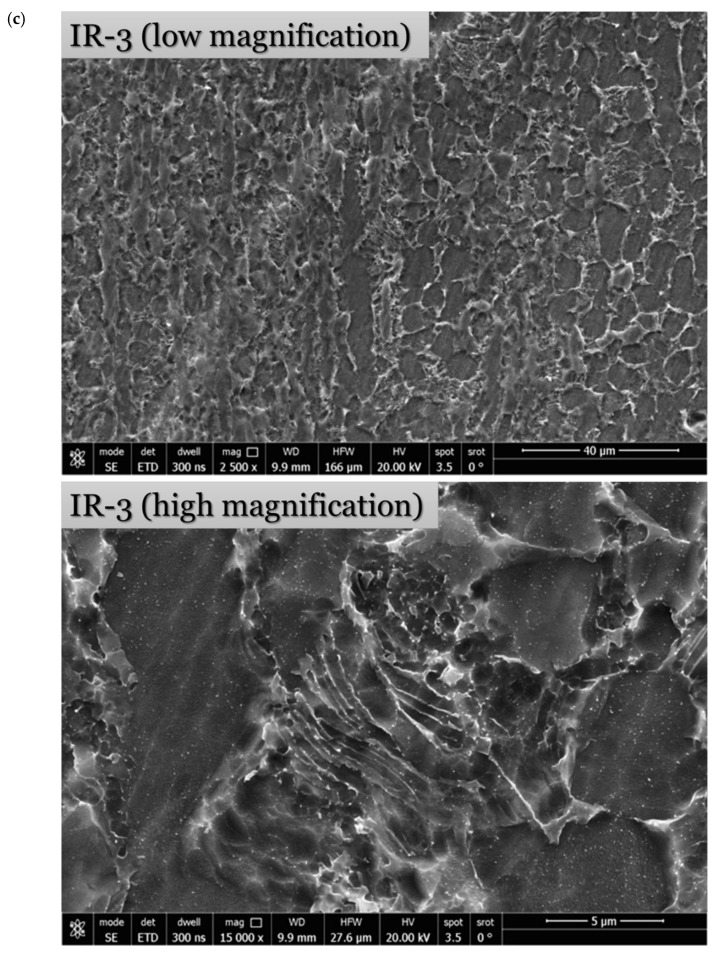
Low- and high-magnification SEM micrographs after isothermal hot-rolling strategies: (**a**) IR-1, (**b**) IR-2, and (**c**) IR-3.

Optical micrographs and the grain size distribution histograms after isothermal hot-rolling strategies are shown in Figure 6. Low- and high-magnification SEM micrographs after isothermal hot-rolling strategies are demonstrated in Figure 7, in order to reveal the influence on large and focused scopes of the microstructure. As manifested, the fraction of β-phase decreases as the deformation temperature decreases from 900 to 750 °C as it transforms to α phase. It corresponds to the phase diagram of Ti-6Al-4V α + β alloy [12].

Upon applying 75% hot defamation at a constant temperature of 900 °C, a combined deformation mechanism of dynamic recrystallization and α spheroidization occurs. The microstructure of the IR-1 strategy (Figure 6a and Figure 7a) reveals fine recrystallized β grains at the boundaries of equiaxed α + β grains along with α spheroids and a finer elongated α phase compared with the as-received microstructure. In dynamic recrystallization, the grain boundary fracture occurs and numerous refined recrystallized β grains appear at the intersection and edges of the grain boundaries. The dynamic recrystallization phenomenon is correlated to thermal activation during thermal deformation, which considers its driving force when it attains a sufficient value because a low strain rate allows for enough deformation time for dynamic recrystallization to occur; hence, the fraction of fine recrystallized grains formed at the grain boundary increases [13]. Relatively heterogeneous distribution of the grain size (Figure 6b) with an average grain size of 6.53 ± 2.75 µm is observed after the IR-1 strategy.

In the IR-2 strategy (Figure 6c and Figure 7b), the alloy is deformed by 75% at a constant temperature of 800 °C. α spheroidization occurs at the expense of the elongated α phase. SEM micrographs reveal fine-lamellar-structure islands. As the deformation temperature decreases to 800 °C, the dynamic recrystallization is excluded as this temperature is lower than the alloy’s recrystallization temperature, which is about 840 °C. As demonstrated from the microstructure and grain size distribution histogram, a finer and more homogeneous microstructure with an average grain size of 6.11 ± 2.06 µm was obtained after isothermal deformation at 800 °C (Figure 6d).

In the IR-3 strategy, the alloy is deformed by 75% at a temperature of 750 °C. The microstructure of IR-3 (Figure 6e and Figure 7c) reveals the formation of coarse α grains with a relatively homogenous grain size distribution, with an average size of 7.32 ± 2.25 µm. SEM micrographs reveal a coarse structure with kinking and bending in the α lamellar. This microstructure configuration is in agreement with Seshacharyulu et al. [14], who found that the structure reveals flow instabilities in the form of adiabatic shear bands and lamellae kinking at low deformation temperatures. It is reported that [2,14], as deformation temperature increases, the intensity of these bands decreases. This is due to the adiabatic conditions that occur upon hot deformation, along with a low thermal conductivity of Ti-6Al-4V alloy that results in weak dissipation of the generated heat during deformation. This locally reduces the flow stress and results in flow localization [2,14].

It is usually known that as the deformation temperature decreases, the average grain size decreases. However, the current results manifest other behaviors. The microstructure after the IR-3 strategy reveals a larger average grain size than the IR-1 and IR-2 strategies. It may be due to the kinetics of the working deformation mechanism. At a low temperature of 750 °C, the spheroidization action decreases; hence, the dominant α phase morphology is coarse elongated α grains.

**Figure 8:**
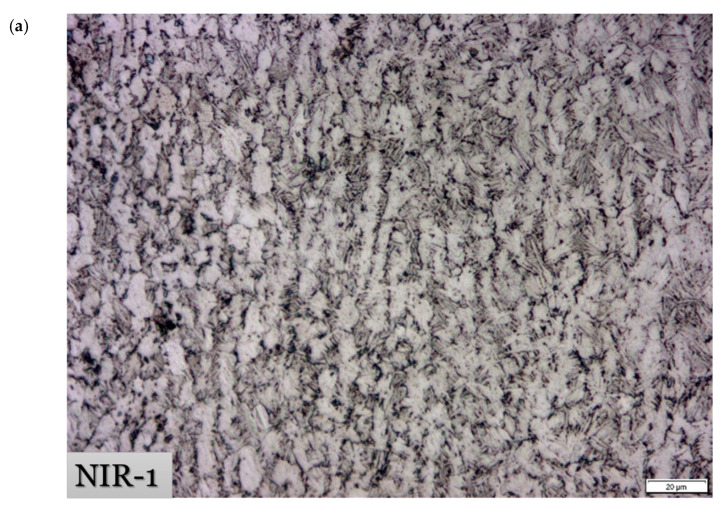

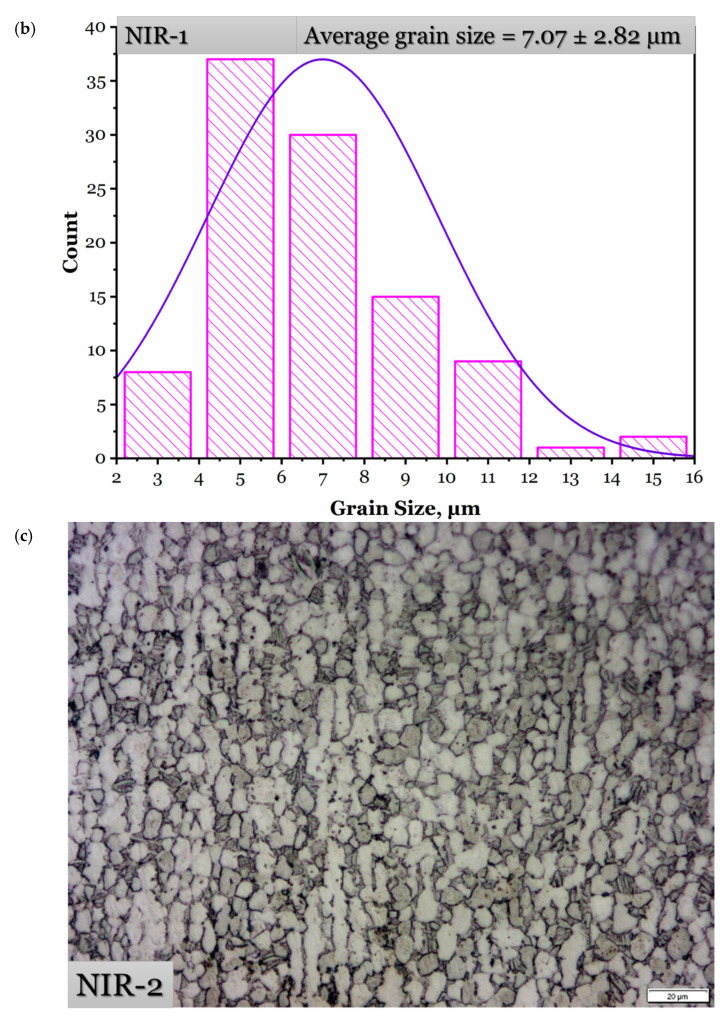

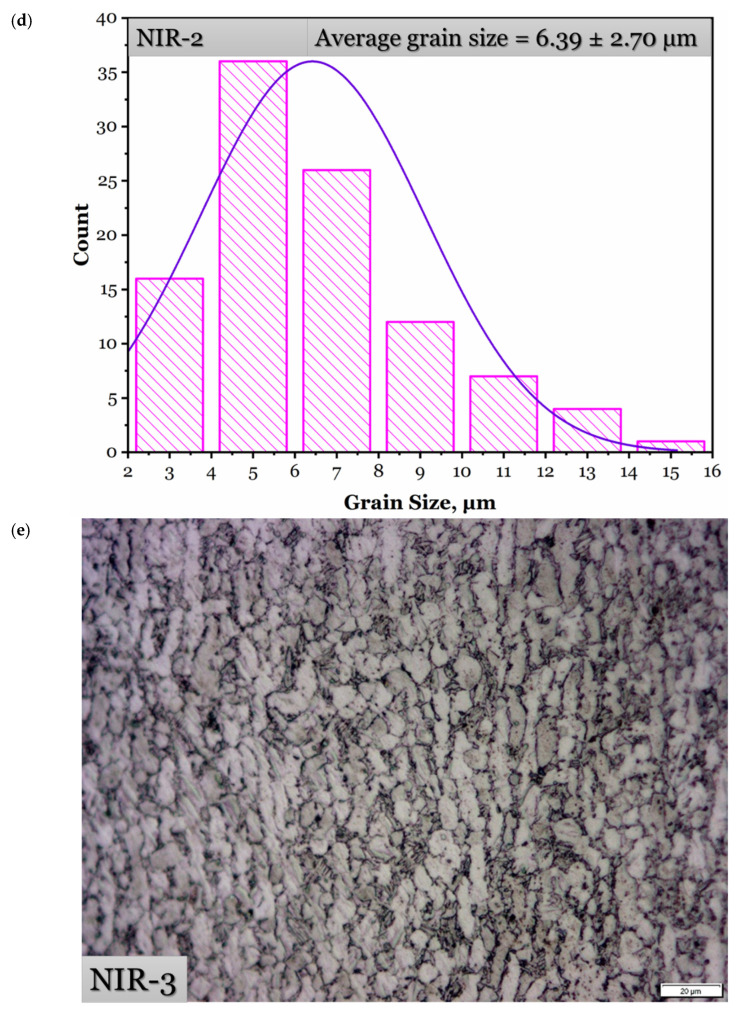

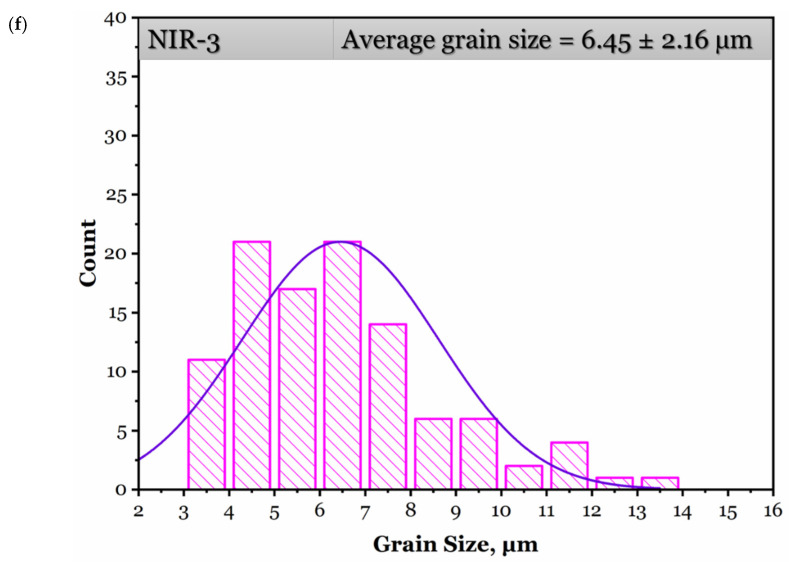
Optical micrographs after non-isothermal hot-rolling strategies and their histograms of the grain size distribution: (**a**,**b**) NIR-1, (**c**,**d**) NIR-2, and (**e**,**f**) NIR-3.

**Figure 9:**
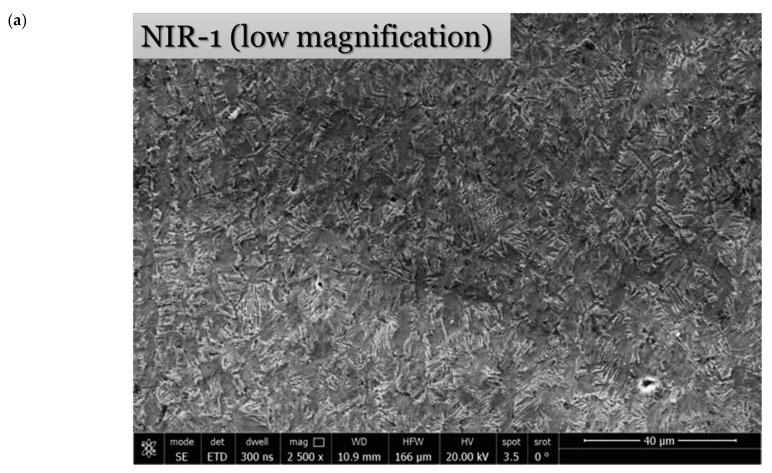

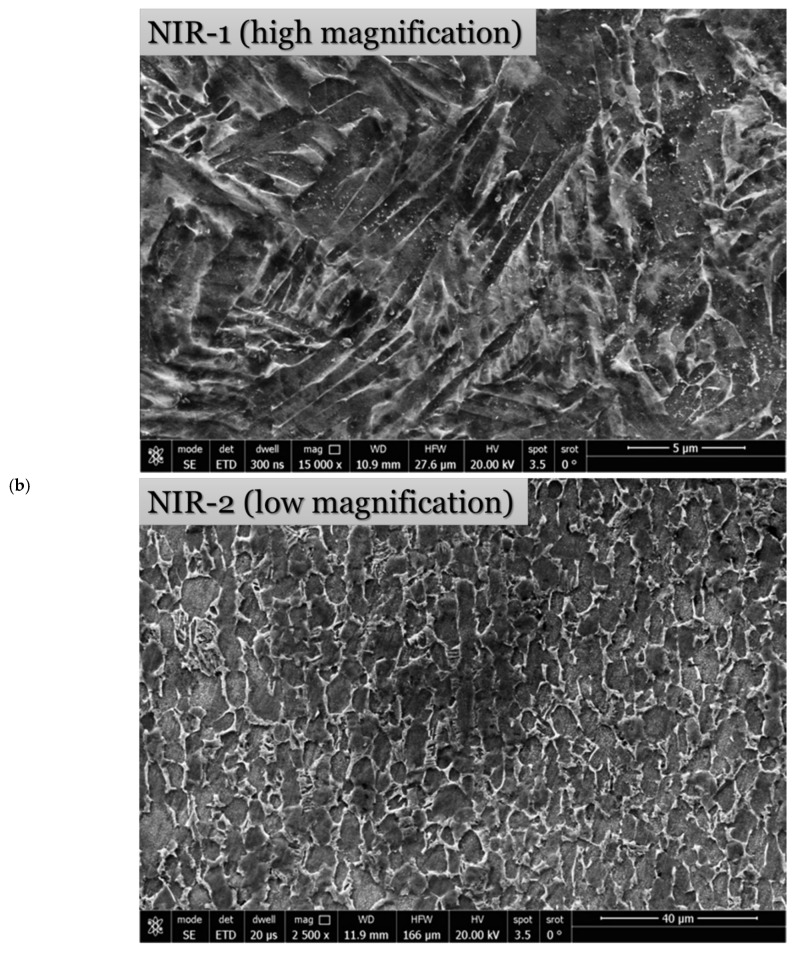

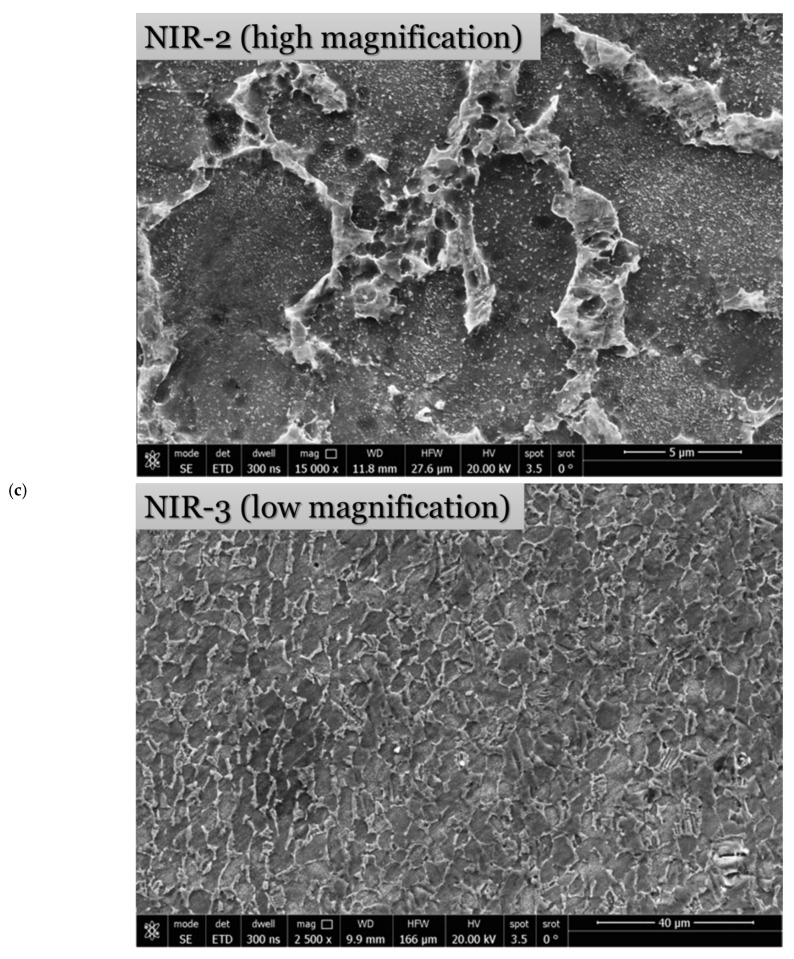

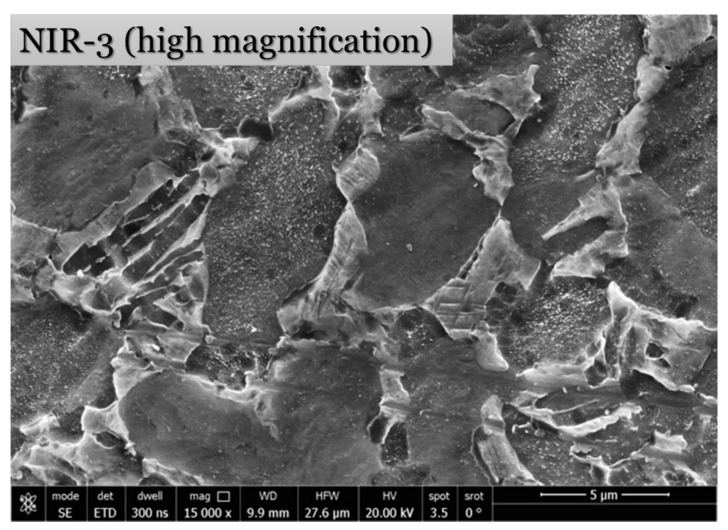
Low- and high-magnification SEM micrographs after non-isothermal hot-rolling strategies: (**a**) NIR-1, (**b**) NIR-2, and (**c**) NIR-3.

**Figure 10:**
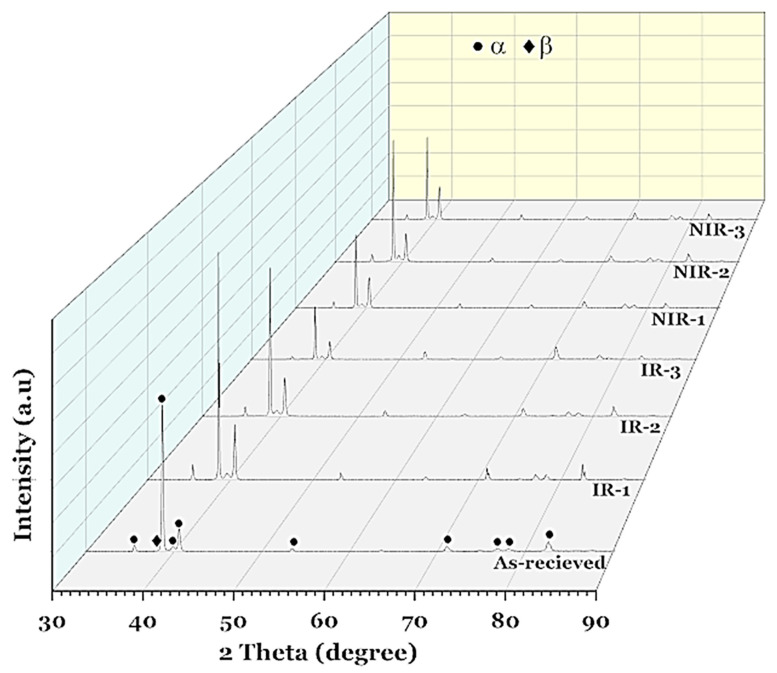
XRD patterns of specimens after the designed hot-rolling strategies.

Optical and SEM micrographs after non-isothermal hot-rolling strategies are shown in Figure 8 and Figure 9, respectively. XRD patterns for specimens after the designed hot-rolling strategies confirm the presence of α and β phases with varying amounts, as shown in Figure 10.

Following the NIR-1 strategy, the alloy is deformed by 50% at 900 °C, followed by cooling to 750 °C and deforming by 25%. The initial microstructure transformed into a bent needle α colony, with coarse and elongated α aggregation (Figure 8a and Figure 9a). A small fraction of the recrystallized β phase is dispersed in the microstructure. This is evidence of the occurrence of dynamic recrystallization upon hot deformation at 900 °C. Then, this recrystallized β phase is transformed into an acicular morphology during cooling to 750 °C. It is reported that [12] increases the deformation temperature, reducing the aspect ratio of the α phase and resulting in a higher amount of spheroidization. This fact is in agreement with the current results. As demonstrated in Figure 8b, a heterogeneous grain size distribution of an average size of 7.07 ± 2.82 µm results after NIR-1. It may be interpreted by the coarsening of α grains upon cooling to 750 °C.

In the NIR-2 strategy, the alloy is heated to 900 °C and soaked for 15 s, then cooled to 850 °C and deformed by 35%, followed by cooling to 800 °C and deformed by 25%. Finally, the alloy is cooled to 750 °C and deformed by 15% with subsequent free cooling. As shown in Figure 8c and Figure 9b, a mixture of spheroidized and elongated α phases is formed. A tiny acicular α colony and a small fraction of recrystallized β phase are also dispersed in the microstructure. Although different morphologies are formed, a more refined heterogeneous grain size distribution of an average size of 6.39 ± 2.70 µm (Figure 8d) is formed compared with the microstructure after the NIR-1 strategy.

In NIR-3, the alloy is deformed by 40% at 900 °C, then cooled to 850 °C and deformed by 20%, followed by cooling to 800 °C and deforming by 10%. Finally, the alloy is cooled to 750 °C and then deformed by 5% with subsequent free cooling. Figure 8e and Figure 9c demonstrate similar microstructure constituents, as produced after the NIR-2 strategy. A higher fraction of acicular α colony and recrystallized β phase is dispersed in the microstructure compared with NIR-2. The average grain size is 6.45 ± 2.16 µm, with relatively homogeneous distribution.

### 3.2. Effect of Hot-Rolling Strategies on the Hot Flow Behavior and Productivity

#### 3.2.1. Hot-Rolling Strategies vs. Flow Behavior

Uniaxial hot compression tests using a Gleeble simulator are used to recognize the flow behavior and characteristics of the material in hot deformation. This technique is a cost-effective tool that can simulate actual industrial processes, aiming to establish relationships between the processing parameters, flow behavior, and final mechanical properties [12]. Accurate characterization of the flow behavior during hot deformation can assist the material designers in optimizing the deformation parameters.

**Figure 11:**
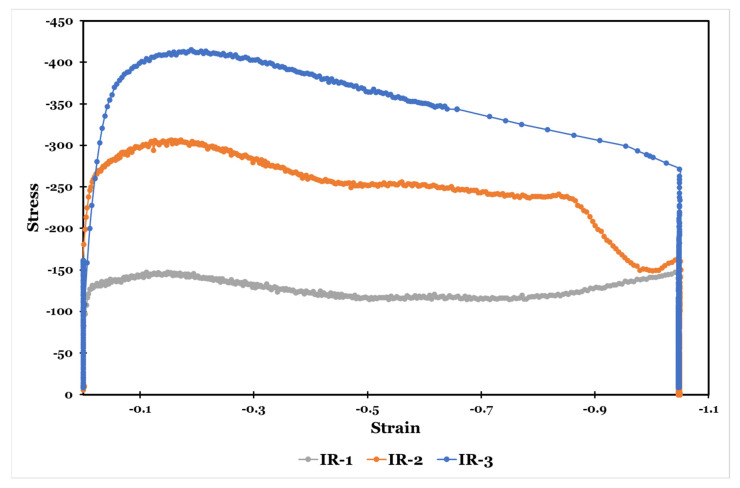
Flow stress-strain curves of the specimens during isothermal hot-rolling strategies at a strain rate of 0.1 s^−1^.

The flow stress-strain curves of the specimens during isothermal hot-rolling strategies (IR), at a strain rate of 0.1 s^−1^, are shown in Figure 11. As manifested, the value of the flow stress is susceptible to the deformation temperature. The flow stress decreases as the deformation temperature increases as a result of softening induced by heating due to atom rearrangement.

Upon deformation in each IR strategy, the flow stress decreases after attaining a peak value. It is attributed to the extensive flow softening that occurs as a result of altering the initial microstructure of the Ti-6Al-4V alloy [13]. The 0.15 strain is required for the beginning of the dynamic softening, which is accomplished by the dynamic recrystallization and/or spheroidization mechanisms.

In the IR-1 strategy, the stress-strain curve becomes flat and then returns to a slight increase. This is attributed to the dynamic balance that happens between the occurring flow softening and work hardening [13].

In IR-2 and IR-3 strategies, the extent of the softening continues to increase on deformation. In the IR-2 strategy, the dynamic softening is mainly due to the spheroidization of the lamellar α phase. This phenomenon cancels the primary work hardening. In the IR-3, kinking and spheroidization of α lamellae and the growth of the equiaxed primary α phase correspond to the continuous flow softening [2,8].

**Figure 12:**
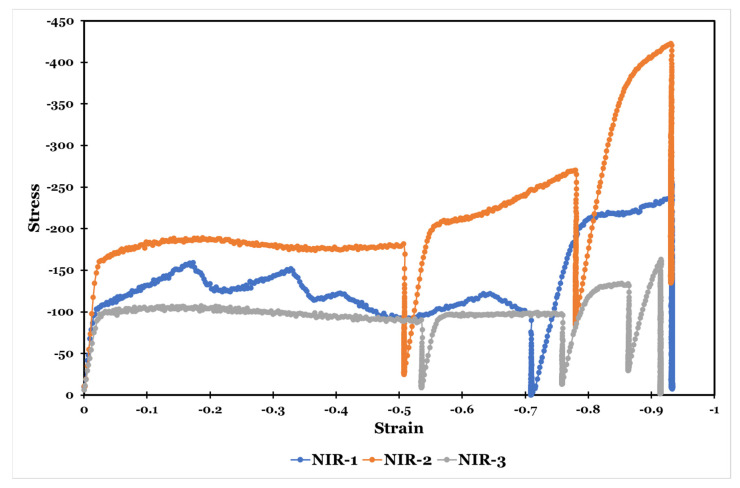
Flow stress-strain curves of the specimens during non-isothermal hot-rolling strategies at a strain rate of 0.1 s^−1^.

Figure 12 shows the flow stress-strain curves of the specimens during non-isothermal hot-rolling strategies at a strain rate of 0.1 s^−1^.

It can be observed that there is little change in the flow stress value in the isothermal hot-rolling strategies at 900 °C compared with the first pass of the NIR-1 and NIR-3 strategies. In NIR-2, the initial flow stress increases as the starting deformation temperature decrease to 850 °C.

During non-isothermal hot-rolling strategies (NIR), upon the initial deformation, the flow stress value increases due to the work hardening until it reaches the peak stress value. As the strain continues to increase, the softening effect of the dynamic recrystallization and spheroidization occurs; hence, the flow stress progressively decreases.

It is observed that the stress-strain curve becomes flat during some passes of NIR strategies and the flow stress is maintained at a constant value. This is attributed to the dynamic balance that happens between the work-hardening and flow-softening phenomena [13]. Other passes show an increase in flow stress during straining. In this case, work hardening is dominant. The observed flow hardening indicates that the deformation mode changes during straining as the hardening becomes prevalent as a result of the grain-growth effect (dislocation slips govern plastic deformation) [15]. It is also observed that the hardening flow increases with decreasing deformation temperature.

Liu et al. [13] demonstrated that, as a result of the lattice friction due to the presence of solute atoms, the increase in the sliding velocity is limited. At the maximum value of the dislocation sliding velocity, the equilibrium occurs. If the strain rate increases at this constant dislocation sliding velocity, the dislocation density sharply increases. Hence, the flow stress increases and work hardening occurs.

In the NIR-1 strategy, the contribution of the formation of acicular α colonies in the softening phenomenon is much more significant than the effect of dynamic recrystallization and/or spheroidization [13]. Drops and oscillations were observed, which indicates the possibility of unstable or localized plastic flow of the material [14].

On the other hand, in the NIR-2 strategy, the flow softening is dominant in the first pass due to the dynamic spheroidization of the lamellar α phase. The other two passes of the NIR-2 strategy show flow hardening, whereas the NIR-3 strategy shows little change in the flow stress upon deformation in the first and second passes. It may be attributed to dynamic recrystallization and flow hardening combined. The third and fourth passes of the NIR-3 strategy manifest slight flow hardening.

#### 3.2.2. Hot-Rolling Strategies vs. Total Strategy Time

The pursuance time of the rolling strategy on the production line must be considered. As a rolling strategy takes longer to perform, it will be more expensive [17]. In the current work, the calculations of the total rolling strategy time are estimated. Table 2 presents the schedule for the designed hot-rolling strategies.

For isothermal hot-rolling strategies, the strategy that takes the shortest processing time is the IR-3 strategy, which requires 512.5 s to be executed, followed by IR-2 and IR-1 strategies, which consume 547.5 and 617.5 s, respectively. For non-isothermal hot-rolling strategies, the NIR-1 strategy takes the shortest processing time of 552.5 s, while NIR-2 and NIR-3 strategies consume the same time of 572.5 s.

Generally, from the standpoint of cost saving, the IR-3 strategy is the most effective in achieving time-saving demand and, hence, promoting productivity, followed by IR-2 and then NIR-1 strategies.

### 3.3. Mechanical Properties and Industrial Performance of the Finished Sheets

**Figure 13:**
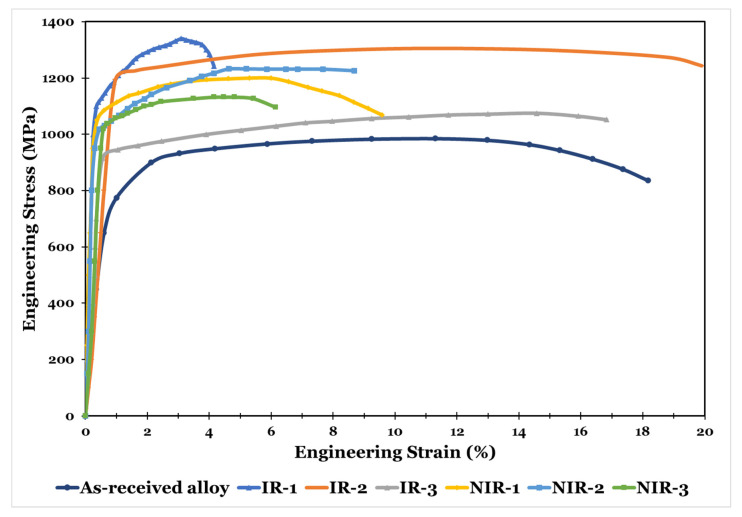
The engineering stress–strain curves of the practically hot-rolled sheets.

The engineering stress-strain curves of the practically hot-rolled sheets are shown in Figure 13. Table 3 summarizes the tensile properties. All the rolling strategies improve the ultimate and yield strength compared with the as-received alloy.

For isothermal hot-rolling strategies, as the deformation temperature decreases in the α + β range from 900 °C to 750 °C, the ultimate tensile strength decreases by 19.7%. This result is attributed to the formation of a high amount of α phase at the expense of the β phase as the temperature decreases. On the other hand, the yield strength does not follow the expected behavior, as the yield strength of the IR-2 strategy is the highest among the isothermal hot-rolling regimes. In the IR-1 and IR-3 strategies, the strength increases at the expense of the ductility compared with the as-received alloy. Only the IR-2 strategy enhances the total elongation by 9.5% compared with the as-received alloy.

The non-isothermal hot-rolling strategies result in an improvement in the strength at a considerable reduction in the ductility. The NIR-2 strategy provides the highest ultimate tensile strength, followed by NIR-1, then NIR-3 strategies.

The strain-hardening exponent and yield strength to ultimate tensile strength ratio are calculated for all specimens (Table 3). The designed hot-rolling strategies have a wide range of strain-hardening exponents and strength ratios. These strategies can be employed to satisfy industrial purposes, as these values have considerable importance when the produced sheet is subjected to sheet metal-forming operations to obtain a final product. The lowest value of strength ratio at a moderate value of n exponent is recommended for this purpose.

Tensile toughness (Table 3), i.e., the area under the stress-strain curve, represents the total amount of energy the sheet can absorb before failure. The higher this value, the higher the ability of the sheet to withstand impact loading before failure. The designed hot-rolling strategies give a wide range of tensile toughness values, which can meet several applications’ needs and desires.

By analyzing the tensile results and connecting them with the industrial purposes and performance, it is found that the IR-1 strategy can be used for producing simple-shape products, utilizing the subsequent sheet metal operations, as the produced sheets exhibit a low strength ratio of 0.843 and a high value of n exponent of 0.08, with low ductility of 3.95% at a relatively high yield strength of 1128.9 MPa. The IR-2 strategy can produce very-simple-shape products by utilizing the subsequent sheet metal operations, whereas the produced sheets exhibit the highest strength ratio of 0.919 and a low value of n exponent of 0.04, with a high ductility of 19.89% at a high yield strength of 1200 MPa. The produced sheet after the IR-2 strategy can be used for high-impact loading applications as it reveals a high tensile toughness of 249.19 J. The IR-3 strategy can be used for producing complex-shape products by utilizing the subsequent sheet metal operations. As the produced sheets exhibit the lowest strength ratio of 0.86 and a moderate value of n exponent of 0.06, with high ductility of 16.83% at a low yield strength of 925 MPa, the produced sheet after the IR-3 strategy can be used for high-impact loading applications as it reveals a high tensile toughness of 168.27 J.

For NIR, the NIR-1 and NIR-2 strategies can be used for producing complex-shape products by utilizing the subsequent sheet metal operations, whereas the produced sheets exhibit the lowest strength ratio, with moderate ductility at relatively low yield strength. After NIR-1 and NIR-2 strategies, the produced sheet can be used for light-impact loading applications as it reveals low tensile toughness.

NIR-3 strategy can be used for producing very-simple-shape products by utilizing the subsequent sheet metal operations, as the produced sheets exhibit a high strength ratio of 0.934 and a moderate value of n exponent of 0.05, with moderate ductility of 6.15% at a relatively low yield strength of 1057.35 MPa. The produced sheet after the NIR-3 strategy can be used for very-light-impact loading applications as it reveals moderate tensile toughness.

Generally, the best combination of the mechanical properties is manifested by the IR-2 strategy, followed by the NIR-2 and NIR-1 strategies.

### 3.4. Optimizing Hot-Rolling Strategy Type

**Figure 14:**
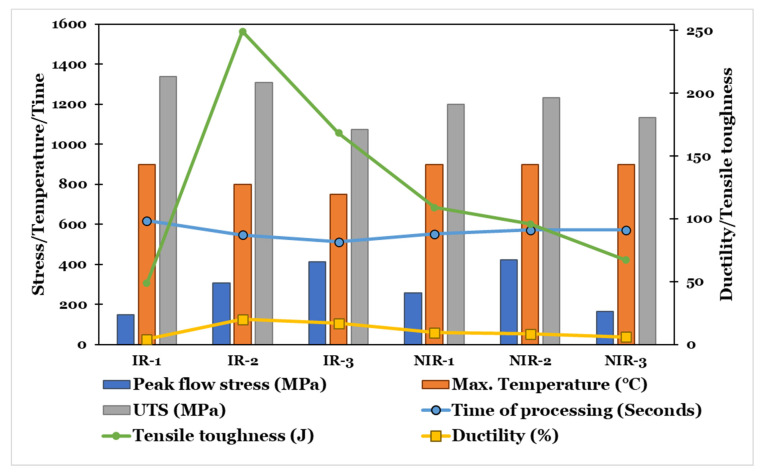
Optimizing hot-rolling strategy type at a strain rate of 0.1 s^−1^.

In order to select the optimal hot-rolling strategy, a relation between the processing parameters (i.e., deformation temperature, flow stress value, and time of processing) and the final mechanical properties and performance (strength, ductility, and tensile toughness) should be correlated, as presented in Figure 14. The optimal hot-rolling strategy should fulfill the criteria of the best combination between the mechanical properties at the most cost-effective processing parameters, i.e., low temperature, low flow stress value, and short time of processing.

As demonstrated by Figure 14, the optimal hot-rolling strategy is the IR-2 strategy, followed by the NIR-2 strategy. These strategies exhibit similar microstructural characteristics. Both reveal a multimodal microstructure that is composed of fine constituents of α spheroids, equiaxed α, and elongated α, with low aspect ratio, acicular α colony, and fine recrystallized β phase. This microstructure results in a good combination of mechanical properties. The worst hot-rolling strategies are IR-1 and NIR-3. IR-3 and NIR-1 strategies show moderate trends.

## 4. Conclusions

In this study, a relationship between microstructural characteristics, flow behavior, mechanical properties, and the different cost-effective processing parameters, i.e., deformation temperature, flow stress value, and time of processing for the Ti-6Al-4V alloy, was developed. The following conclusions can be drawn:

1. Grain size of the as-received semi-finished hot-rolled plate is slightly affected after isothermal and non-isothermal hot-rolling strategies in the α + β range to produce the final sheet product, whereas the morphology and distribution of the phases are considerably affected.
2. The dynamic flow softening is dominant in all IR strategies after the flow stress attains the peak at a low strain value. Then, dynamic flow softening occurs due to the dynamic recrystallization and α phase spheroidization. While a combination of flow softening and hardening takes place on the different passes of the NIR strategies.
3. The optimal microstructure that fructifies the best combination of mechanical properties is composed of fine constituents of α spheroids, equiaxed α, elongated α with low aspect ratio, acicular α colony, and fine recrystallized β phase.
4. The optimal hot-rolling strategy that fulfills the criteria of the best combination of mechanical properties at the most cost-effective processing parameters is the IR-2 strategy, followed by the NIR-2 strategy. The IR-1 and NIR3 strategies manifest low mechanical and economic impact.
5. The finished sheets produced by the IR-2 strategy can be used for producing very simple-shape products, utilizing subsequent sheet metal operations, and can be used for heavy-impact loading applications. In contrast, the finished sheets produced by the IR-3 strategy can be used to produce complex-shape products for heavy-impact loading applications by utilizing subsequent sheet metal operations.
6. The finished sheets produced by NIR-1 and NIR-2 strategies can be used for producing complex-shape products, utilizing subsequent sheet metal operations, at a moderate toughness for light-impact loading applications.

## Figures and Tables

**Table 1:** Chemical composition (wt%) of the studied hot-rolled Ti-6Al-4V alloy.

Element	Al	V	Fe	C	O	N	H	Ti
wt%	5.89	4.2	0.2	0.1	0.2	0.0016	0.0053	Balance

**Table 2:** The schedule for the different hot-rolling strategies.

Rolling Strategy	Temperature (°C)	Deformation/Pass (%)	Time (S)
**IR-1**	Heating 0–900	0	180
	Soaking at 900	0	15
	1st stroke at 900	75	7.5
	Cooling 900–70	0	415
	**Total**	**75**	**617.5**
**IR-2**	Heating 0–800	0	160
	Soaking at 900	0	15
	1st stroke at 800	75	7.5
	Cooling 800–70	0	365
	**Total**	**75**	**547.5**
**IR-3**	Heating 0–750	0	150
	Soaking at 750	0	15
	1st stroke at 750	75	7.5
	Cooling 750–70	0	340
	**Total**	**75**	**512.5**
**NIR-1**	Heating 0–900	0	180
	Soaking at 900	0	15
	1st stroke at 900	50	5
	Interpass time	0	10
	2nd stroke at 750	25	2.5
	Cooling 750–70	0	340
	**Total**	**75**	**552.5**
**NIR-2**	Heating 0–900	0	180
	Soaking at 900	0	15
	Cooling 900–850	0	10
	1st stroke at 850	35	3.5
	Interpass time	0	10
	2nd stroke at 800	25	2.5
	Interpass time	0	10
	3rd stroke at 750	15	1.5
	Cooling 750–70	0	340
	**Total**	**75**	**572.5**
**NIR-3**	Heating 0–900	0	180
	Soaking at 900	0	15
	1st stroke at 900	40	4
	Interpass time	0	10
	2nd stroke at 850	20	2
	Interpass time	0	10
	3rd stroke at 800	10	1
	Interpass time	0	10
	4th stroke at 750	5	0.5
	Cooling 750–70	0	340
	**Total**	**75**	**572.5**

**Table 3:** Mechanical properties for practically hot-rolled sheets for various hot-rolling strategies.

Rolling Strategy	UTS (MPa)	YS (MPa)	El. (%)	YS/UTS Ratio	Strain-Hardening Exponent (n)	Tensile Toughness (J)
As-received alloy	985.21	712.5	18.17	0.723	0.06	154.19
IR-1	1339.78	1128.9	3.95	0.843	0.08	48.76
IR-2	1304.6	1200	19.89	0.919	0.04	249.19
IR-3	1075.19	925	16.83	0.860	0.06	168.27
NIR-1	1200.86	1080	9.57	0.899	0.05	109.17
NIR-2	1232.1	1030.9	8.48	0.837	0.09	95.99
NIR-3	1132.23	1057.35	6.15	0.934	0.05	67.29
